# Enhancing Wettability of Cu_3_P/Cu Systems through Doping with Si, Sn, and Zr Elements: Insights from First Principles Analysis

**DOI:** 10.3390/ma16062492

**Published:** 2023-03-21

**Authors:** Shimeng Yu, Fang Cheng, Lian He, Weigang Tang, Yongsheng Wang, Rong Chen, Chenglu Hu, Xiao Ma, Hangyan Shen

**Affiliations:** 1 College of Materials and Chemistry, China Jiliang University, Hangzhou 310018, China; 2 Zhejiang Zhekan Testing Co., Ltd., Ningbo 315033, China; 3 Hangzhou Huaguang Advanced Welding Materials Co., Ltd., Hangzhou 311112, China; 4 College of Materials Science and Engineering, Taiyuan University of Technology, Taiyuan 030024, China

**Keywords:** wettability, first principles calculations, work of adhesion (*W_ad_*), charge density differences, the density of states (DOS)

## Abstract

Explaining the wetting mechanism of Cu–P brazing materials and Cu remains challenging. This fundamental research aims to reveal the wettability mechanism of Si, Sn, and Zr doping on the interfacial bond strength of the Cu_3_P/Cu system through the first principles study. We carried out several sets of calculations to test the validity of the result; included in the work are those used to establish the interfacial structure and to analyze the effect of doping on the wettability. Specific analysis was carried out in terms of three aspects: the work of adhesion ($W_{ad}$), the charge density difference, and the density of states (DOS). The calculated results show that doping with Si, Sn, and Zr elements can effectively improve the wettability within the CuP/Cu interface with very high accuracy, and is particularly effective when doped with Zr. These results provide an insightful theoretical guide for enhancing the CuP/Cu system’s wettability by adding active elements.

## 1. Introduction

Copper–phosphorous (Cu–P) brazing alloys are types of brazing filler metal that are primarily composed of copper (Cu) and phosphorus (P), and that are widely applied in the aerospace, electronics, energy, transportation, military, and automobile industries due to their excellent properties [1,2]. P element can decrease the filler metal’s melting temperature and increase the substrate’s spreading area [3]. However, Cu–P solder contains a lot of Cu_3_P compounds, which results in poor plasticity at room temperature. It significantly affects the application of Cu–P solder [4]. Therefore, maintaining the existing advantages of Cu–P brazing materials, while considering the two objectives of cost reduction and performance enhancement, has become one of the hot spots for brazing materials.

Copper–phosphorous (Cu–P) brazing alloys are frequently used in applications where brazing between copper and copper alloys occurs. During the brazing process, the filler metal is melted and flows between the two parts to be joined. The molten filler metal wets the surfaces of the parts, forming a strong metallurgical bond when it solidifies. The wettability of the brazing material can reflect the brazing material’s ability to fill the seam, and one of the most effective ways to improve the wettability between Cu and CuP is to introduce alloying elements into the brazing material. The melting point of the Sn element is 231.93 °C and less expensive. Adding the Sn element into copper–phosphorus brazing material can significantly reduce its melting point and improve its fluidity [5]. Wu [6] shows that adding Sn can increase the spreading area of Cu-7P-1Ag-XSn on the H62 brass plate. The results of Zhang [7] et al. show that the adhesive strength of the Ag/B-terminal ZrB_2_ interface structure is significantly increased by adding the active element Zr.

First principles analysis is a robust computational tool used in materials science and cohesive physics for predicting the properties of materials based on their electronic and atomic structure. First principles calculations based on density functional theory (DFT) are an essential auxiliary approach to the analysis of interface interactions by studying interface structure, work of adhesion ($W_{ad}$), interface energy, energy bands, and electron density at the atomic level [8,9,10]. To improve the wettability of Cu–P brazing materials on the Cu substrate, the effect of the addition of Si, Sn, and Zr on the wettability within the Cu_3_P/Cu system was studied by first principles calculation.

## 2. Computation Methods and Models

### 2.1. Computation Methods

All computations in this paper are performed within the framework of the density functional theory (DFT), employing the projection-enhanced wave method, which is implemented in the Vienna Atomic Simulation Package (VASP) code. The generalized gradient approximation (GGA) is applied to the exchange–correlation function in Perdew–Burke–Ernzerhof (PBE) formalism [11]. The kinetic cut-off energy of the plane wave basis is 500 eV, and the convergence criterion of 10^−8^ eV is adopted. All combinations must meet the current convergence threshold of 0.001 eV∙Å^−1^. Relaxation and total energy are calculated using Gamma-center grids with a k-spacing of about 0.2 Å^−1^ for all structures, including bulks, surfaces, and interfaces. The atomic position and the lattice constant are wholly relaxed for bulks, whereas only the atomic position is completely relaxed for the surfaces and interfaces. The long interaction in calculating periodic boundary conditions is eliminated by utilizing a vacuum space of 12 Å along the c-axis.

### 2.2. Computation Methods

The lattice constants of Cu_3_P (P63CM, No.185) and Cu (Fm-3m, No.225) were calculated and compared with the experimental and database values. The calculated lattice constants of Cu_3_P and Cu are compared in Table 1. The calculation error is less than 1%, which agrees with the experimental and database values.

According to Zhao’s experimental results, the calculation should be performed using the (100) face of Cu_3_P. Therefore, (100) aspects of the Cu_3_P system are concentrated on in this study [15]. According to Bramlett’s lattice mismatch theory [16], the Cu (100) surface can better match the Cu_3_P (100) surface. The Cu (100) and Cu_3_P (100) surfaces are cut from their corresponding bulks.

To balance computational accuracy and hardware resources, convergence tests on Cu (100) and Cu_3_P (100) surfaces of different thicknesses to determine the minimum number of atomic layers. In thermodynamics, surface energy ($\gamma_{s}$), the energy required to cut a surface from a crystal, is generally considered a criterion for surface stability [7]. For the stoichiometric Cu (100) surface model, the surface energy can be obtained through Formula (1) [8].

(1) $$\gamma_{s}=\left(E_{slab}^{relaxed}-NE_{bulk}\right)/2A$$

where $E_{slab}^{relaxed}$ is the total energy of surface structure after relaxation, $E_{bulk}$ is the total energy per atom in bulks, N is the number of Cu atoms in the slab, and A is the area of slab surface considered.

After complete atomic position relaxation, the variation in the surface energy with slab thickness is calculated by Formula (1). The calculated results of the surface energy for different layers are shown in Table 2.

The results show that the slab on the Cu (100) surface of the five atomic layers is thick enough to exhibit bulk-like interiors without computational burden. To maintain symmetry with the number of layers on the Cu_3_P (100) surface, the Cu (100) surface of the six atomic layers was chosen. The Cu (100) surface of the six atomic layers calculated a value of 1.433 J/m^2^, which is consistent with other calculation results of 1.52 J/m^2^ [17]. However, the calculated value is slightly lower than the orientation-averaged experimental value of 1.78 J/m^2^ [18].

**Figure 1:**
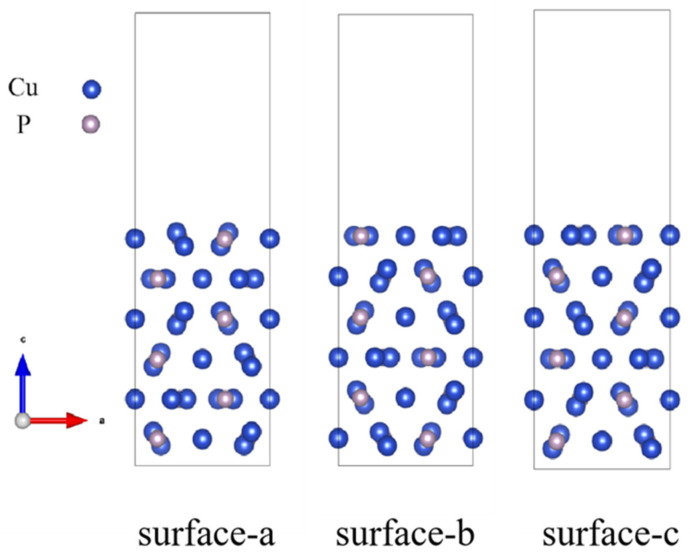
Three different terminations for the Cu_3_P (100) surface.

A surface energy calculation is performed for the non-stoichiometric Cu_3_P (100) surfaces model; this is to confirm the most stable termination for the (100) facet. As shown in Figure 1, three different terminations for the Cu_3_P (100) surface are obtained by cleaving the geometric optimized bulk Cu_3_P structure. The surface energy ($\gamma_{s}$) of these three surfaces was calculated using the following formula [19]:

(2) $$\gamma_{s}=\frac{1}{2A}\left[E_{slab}^{unrelax}-nE_{bulk}\right]+\frac{1}{A}\left[E_{slab}^{relax}-E_{slab}^{unrelax}\right]$$

where $\gamma_{s}$ is the surface energy; $E_{slab}^{unrelax}$ and $E_{slab}^{relax}$ are the total energy of the unrelaxed and relaxed slab on the surface, respectively; $E_{bulk}$ is the energy of a bulk Cu_3_P formula unit; n is the number of Cu_3_P units in the slab; and A is the surface area of the slab.

The computed surface energies of surface a, surface b, and surface c are 0.4376 J/m^2^, 0.5614 J/m^2^, and 0.5611 J/m^2^, respectively. Note that the surface structure with lower surface energy is more stable. Comparatively speaking, the trend of surface stability is surface a > surface c > surface b, which indicates that surface a is the most stable surface of the three (100) terminations, which is consistent with Zhao’s calculation [15]. Therefore, only surface a is considered for subsequent calculations.

The lattice constants of Cu_3_P (100) (11) are a = 6.974 Å, b = 7.199 Å, and Cu surfaces slab a = b = 7.705 Å, respectively. When calculating the interface energy and the separation work, the energy between the interface and the two separated surfaces cancel each other out. The influence of artificial lattice strain on the calculation of interface stability can be eliminated to the greatest extent [20]. The degree of interface lattice mismatch caused by the introduced lattice strain can be calculated from Formula (3):

(3) $$\alpha =(a_{2}-a_{1})/a_{2}\times 100\%$$

where $a_{2}$ and $a_{1}$ are the lattice constants of the interface and surface structure, respectively. The lattice constant of the interface model is defined as the average values of the lattice constants of Cu (100) and Cu (100) surfaces, which are a = 7.340 Å, b = 7.452 Å. The lattice fitness is 4.986% < 5%, which accords with the ideal interface model.

**Figure 2:**
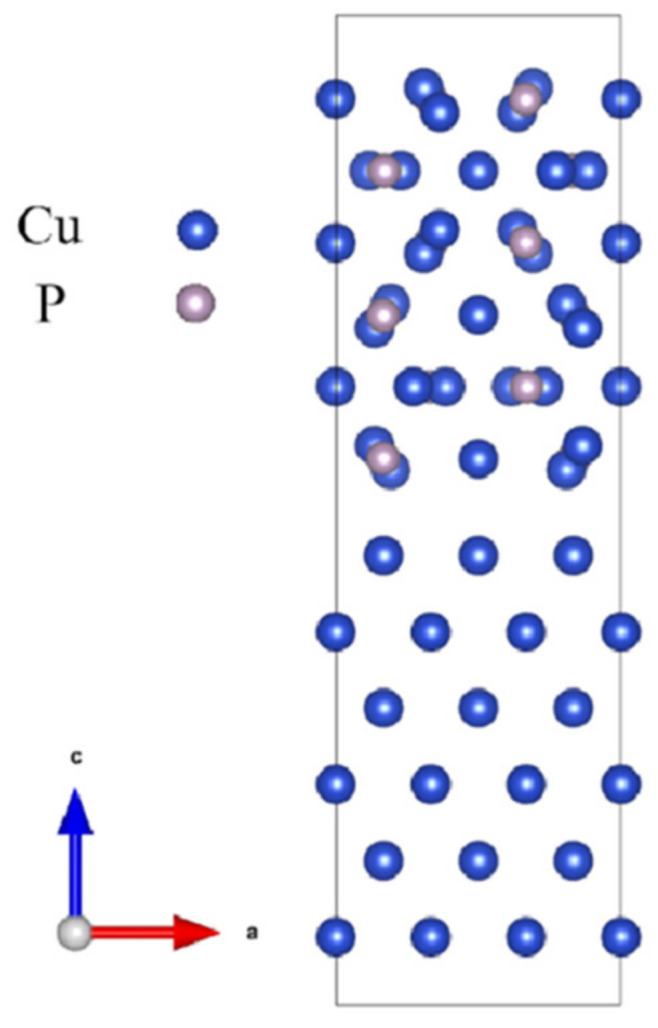
The interface model of Cu_3_P/Cu.

Considering the high thermal stress in the existing Cu_3_P/Cu wetting system, the lattice parameters on the Cu surface are compressed, and the lattice parameters on the Cu_3_P surface are stretched. The interface model is shown in Figure 2.

## 3. Results

### 3.1. Work of Adhesion and Interface Stability of Cu_3_P/Cu Interface

Wettability is the ability of a liquid to spread itself on a solid surface, which can be characterized by the wettability angle and work of adhesion ($W_{ad}$). The wettability angle (θ) can be defined by Young’s equation [21]:

(4) $$cos\theta =\frac{\sigma_{sv}-\sigma_{sl}}{\sigma_{lv}}$$

where $\sigma_{sv}$, $\sigma_{sl}$, and $\sigma_{lv}$ are the interface energies of solid–vapour, solid–liquid and liquid–vapor, respectively. θ  is the wetting angle between the liquid and solid.

Work of adhesion ($W_{ad}$) is a thermodynamic measure of wettability, and it represents the work required to separate the liquid/solid interface per unit area, and the surface energy of the solid and the liquid. In addition to the energy consumed at the liquid/solid interface, the excess is converted into work of adhesion ($W_{ad}$) [21].

(5) $$W_{ad}=\sigma_{sv}+\sigma_{lv}-\sigma_{sl}=\sigma_{lv}\left(1+cos\theta \right)$$

According to Formula (5), the work of adhesion ($W_{ad}$) is inversely proportional to the wetting angle (θ). When the value of the work of adhesion ($W_{ad}$) is raised, the wettability is correspondingly increased.

To evaluate interface bonding, we use first principle calculations to obtain the work of adhesion ($W_{ad}$) derived from the following formula [22].

(6) $$W_{ad}=\left(E_{Cu_{3}P}^{slab}+E_{Cu}^{slab}-E_{Cu_{3}P/Cu}^{slab}\right)/A$$

where $E_{Cu_{3}P}^{slab}$ and $E_{Cu}^{slab}$ are the total energy of the relaxed Cu_3_P (100) and Cu (100) slabs, respectively, and $E_{Cu_{3}P/Cu}^{slab}$ is the total energy of the relaxed Cu_3_P (100)/Cu (100) interface.

**Figure 3:**
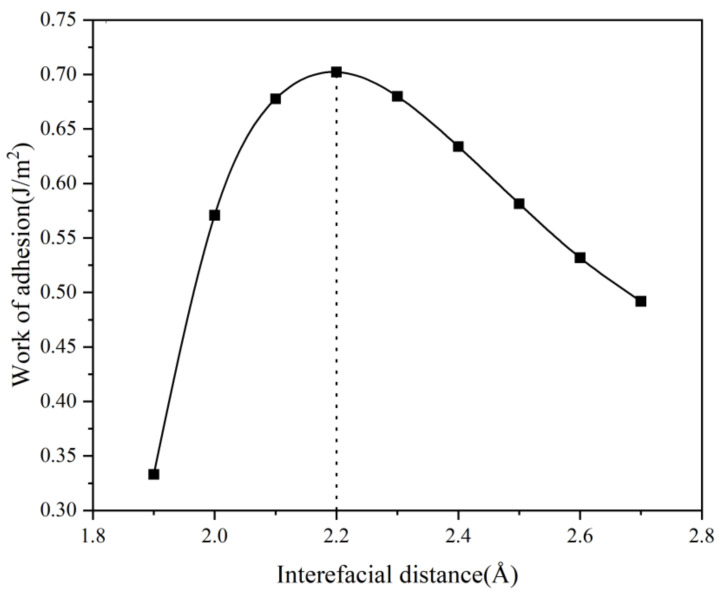
The relationship between the unrelaxed segregation distance ($d_{0}$) and the work of adhesion ($W_{ad}$).

For the interface model, the proper segregation distance ($d_{0}$) dramatically affects the work of adhesion ($W_{ad}$) and the interface atomic bond. Therefore, the work of adhesion ($W_{ad}$) should be calculated at different segregation distances ($d_{0}$) before the structural optimization. Figure 3 shows the relationship between the unrelaxed segregation distance ($d_{0}$) and the work of adhesion ($W_{ad}$) calculated value, which is based on the universal binding-energy relation (UBER) [23]. From Figure 3, the peak of the curve represents the optimal initial separation distance ($d_{0}$) corresponding to the maximum, and the optimal separation distance ($d_{0}$) corresponding to the relaxed geometry can be obtained in the following calculations.

### 3.2. CuP–(Si, Sn, and Zr)/Cu Interfaces

#### 3.2.1. Interfacial Atomic Structure

For simplicity, by substituting one site Cu or P atom in bulk Cu_3_P, the formation energy of doped Si, Sn, and Zr atoms can be calculated by Formula (7) [24], and the calculated results are shown in Table 3.

(7) $$E=E_{total}-E_{Cu_{3}P}+aE_{x}-aE_{y}$$

where $E_{total}$ is the total energy of the doped system, $E_{Cu_{3}P}$ is the total energy of bulk Cu_3_P, $E_{x}$ and $E_{y}$ are the energy of the free atoms to be replaced, respectively, and a is the number of atoms to be replaced.

From Table 3, for simplicity, only one substituted site in the Cu_3_P (100)/Cu (100) interface structure is considered in the following calculation. The doping formation energy of Si and Sn atoms replacing P atoms is lower than that of Cu atoms, and the doping formation energy of Zr atoms replacing Cu atoms is lower than that of P atoms.

**Figure 4:**
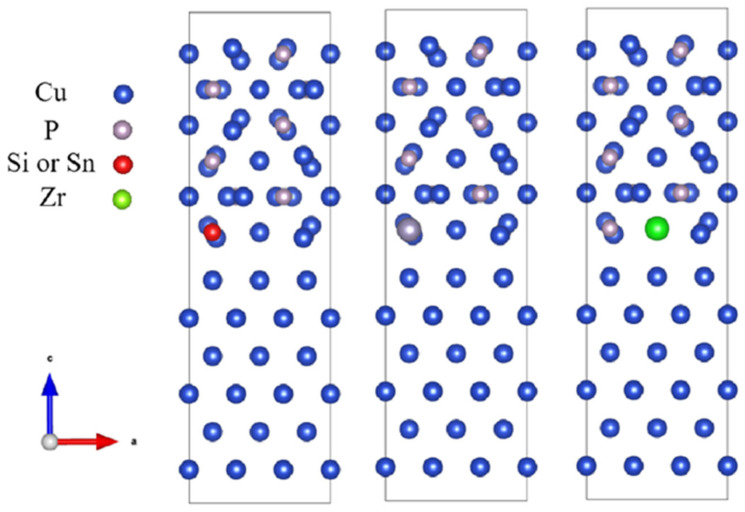
The doped CuP-X/Cu interface model.

The doped CuP-X/Cu interface model is shown in Figure 4. In the wetting model, the elements (Si, Sn, and Zr) of 2.08% were added into Cu_3_P. Thus, the two Cu_3_P (100) interface and (3 × 3 × 1) Cu (100) supercells (102 atoms) were constructed, where an element atom substituted only one atom at different layers.

**Figure 5:**
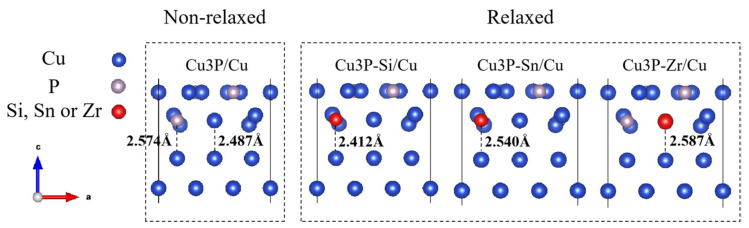
Local deformation of the atomic interface structure induced by elements (Si, Sn, and Zr) before and after interfacial relaxation of CuP/Cu.

Figure 5 shows the local deformation of the relaxed and unrelaxed atomic interface structure induced by elements (Si, Sn, and Zr) in the CuP/Cu interface. There is a slight change in the atoms of the doped interface after geometric optimization. The Si-Cu and Sn-Cu bond lengths in the interface are 2.412 Å and 2.540 Å, respectively, which are smaller than before geometric optimization, and the Zr-Cu bond lengths in the interface are 2.587 Å, which is more significant than before geometric optimization. It is worth noting that these bond lengths are slightly less than the sum of the covalent radii of the corresponding atoms, due to the overlap of wave functions between the interface atoms [8].

#### 3.2.2. Work of Adhesion

**Figure 6:**
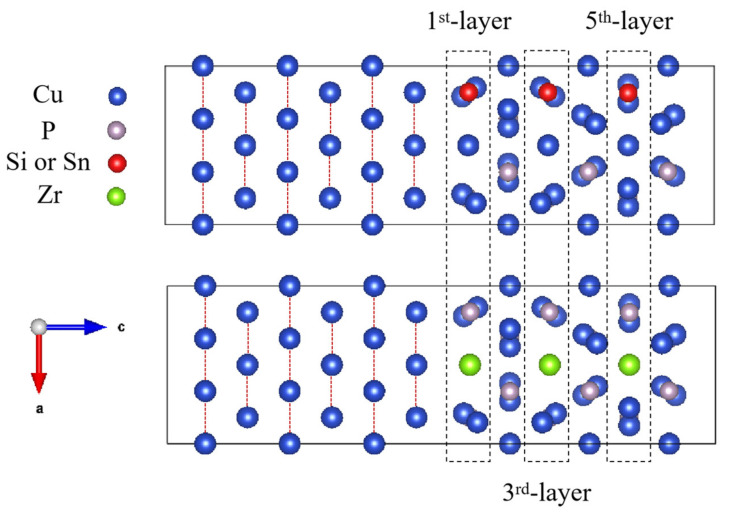
Schematic of the elements (Si, Sn, and Zr) doped at different layers in CuP/Cu interface structure.

**Figure 7:**
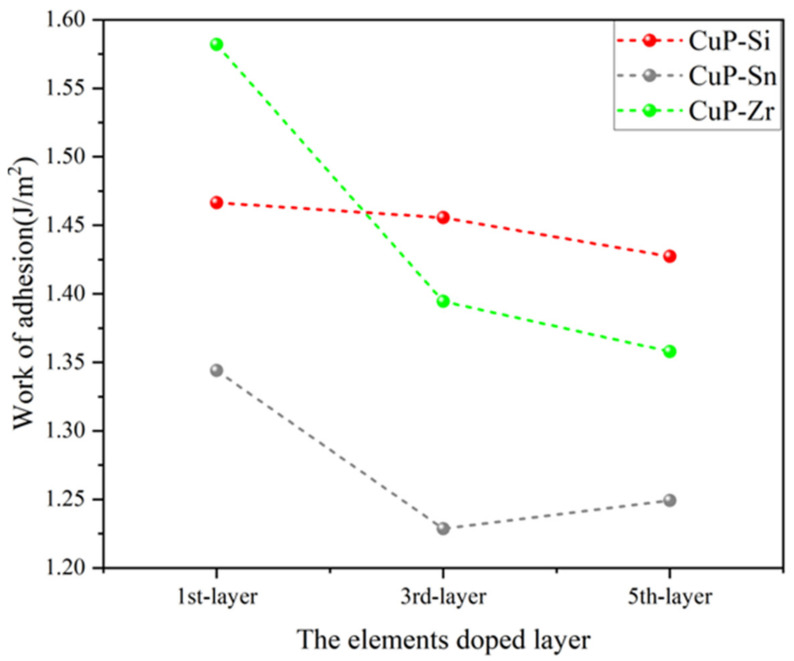
The work of adhesion ($W_{ad}$) of the elements (Si, Sn, and Zr) doped at different layers in CuP/Cu interface structure.

Figure 6 shows the schematic of the elements (Si, Sn, and Zr) doped at different layers in the CuP/Cu interface structure, and Figure 7 shows the work of adhesion ($W_{ad}$) of the doped CuP-X/Cu interfaces with the different elements substituting one Cu or P atom at different layers. The elements doped in the first layer can be significantly enhanced by work of adhesion ($W_{ad}$), and this effect is greatly diminished when the element is located in another layer away from the interface. It is shown that the doped element in the first layer can induce a strong driving force to move it from the inner CuP slab to the interface. Compared with the work of adhesion ($W_{ad}$) of the doped CuP-X/Cu interfaces (Table 4), the Zr elements doped in the interface of CuP/Cu give rise to sharp enhancements in the work of adhesion ($W_{ad}$) from 1.2869 to 1.5819 J/m^2^. After doping Si and Sn, the work of adhesion ($W_{ad}$) at the interface increases slightly to 1.4273 and 1.3440 J/m^2^, respectively. The results show that the interface bonding strength of the CuP/Cu interface structure can be significantly increased by doping Si, Sn, and Zr elements. Thus, the wettability can be improved by doping Si, Sn, and Zr elements in the CuP/Cu interface structure model.

#### 3.2.3. Electronic Properties of the Interface

It is well-known that good interface bonding involves high strength and stability, usually derived from good wettability for the reactive and non-reactive wetting models. It is closely related to the interface’s chemical bonding (including covalent, metallic, and ionic bonds) [7].

**Figure 8:**
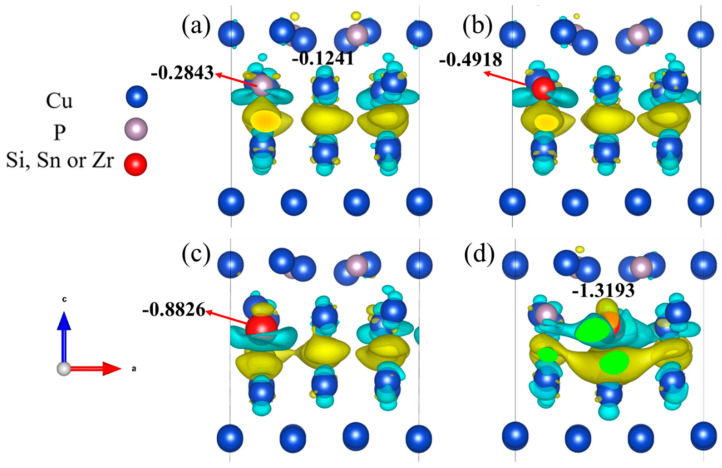
Schematic of the three-dimensional charge density differences of CuP-X/Cu interface: (**a**) CuP/Cu; (**b**) CuP-Si/Cu; (**c**) CuP-Sn/Cu; (**d**) CuP-Zr/Cu.

The charge density difference is calculated to better understand the interface interaction between the CuP-X alloys and Cu substrate. The aim is to reveal the ionic bonding between interfacial atomic layers between CuP and Cu before and after the addition of Si, Sn, and Zr elements, as shown in Figure 8. The yellow and blue areas represent enrichment and the loss of electrons in the schematic of charge density difference. The charge depletion around Si, Sn, and Zr atom can be observed in the interface structure after doping (Figure 8a–c), which plays an essential role in forming Si–Cu, Sn-Cu, and Zr-Cu ionic bonds. The quantitative analysis of border partial charge can also reflect this conclusion. Compared with the interface structure before doping (Figure 8a), the charge redistribution between interface atoms becomes more robust after doping. Thus, this results in relatively muscular bonding strength between CuP-X and Cu, indicating that the wettability within the interface increases correspondingly, which is consistent with the calculation of the work of adhesion ($W_{ad}$) in the previous section.

**Figure 9:**
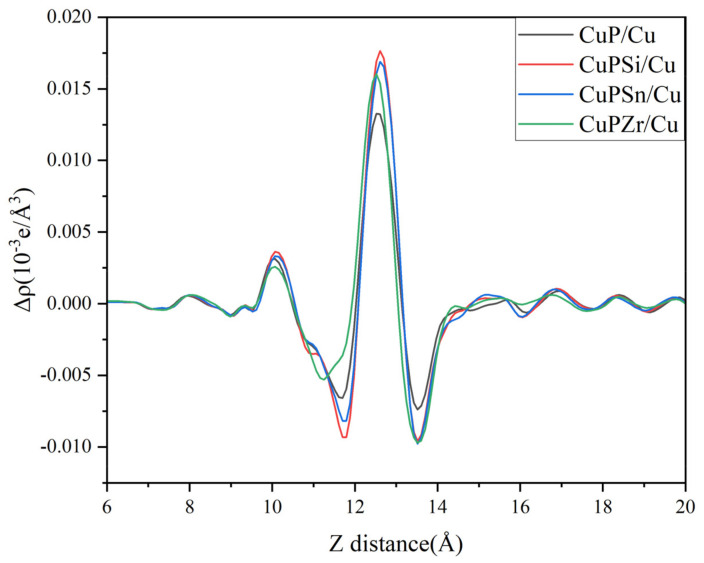
The plane-average electron difference of CuP-X/interface.

The plane-average electron difference of the CuP-X/Cu interface reflects the quantitative information on charge transfer, as shown in Figure 9. Compared with the Cu_3_P/Cu interface, there is more obvious charge accumulation in the interface after adding Si, Sn, and Zr, and the charge loss near the CuP-X side is also more obvious, which proves the trend of CuP-X transfer to the interface.

**Figure 10:**
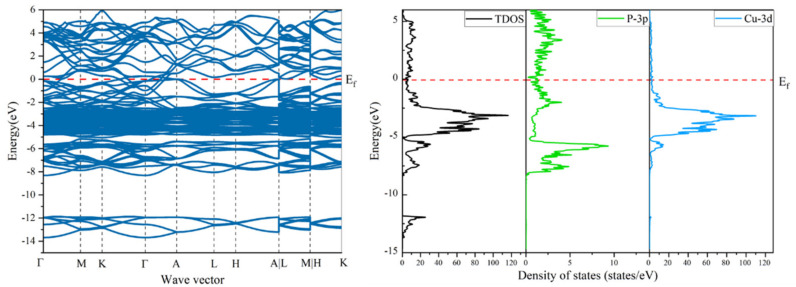
Calculated band structure and density of states of bulk Cu_3_P.

To further illustrate the wettability mechanism between Cu alloys and before and after doping on the surface of CuP, the total density of states (TDOS) and projected density of states (PDOS) of Cu_3_P, CuP-Si/Cu, CuP-Sn/Cu, and CuP-Zr/Cu interfaces were obtained. Firstly, the band structure and density of states of bulk Cu_3_P were calculated, as shown in Figure 10. The result of the calculation is close to the result of the previous calculation. Generally, the same electronic structure can be visualized in two different ways: DOS and band structure. Electrons from both the Cu-3d and P-3s states contribute to DOS. The 3s orbital electrons of the P atom on bulk Cu_3_P majorly occupy the −14 eV~−12 eV region.

In contrast, 3d orbital electrons of the Cu atom mainly occupy the −5 eV~0 eV region. The band range is relatively narrow, indicating that the 3d orbitals of Cu are localized, which is a common feature of transition metals in general.

**Figure 11:**
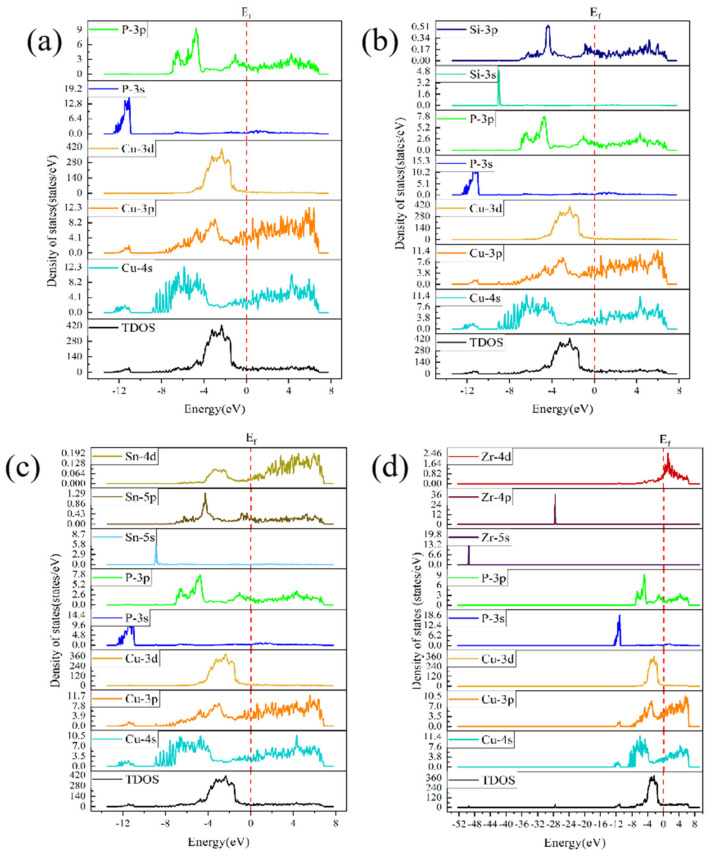
Schematic of the changes in projected DOS (PDOS) of (**a**) CuP/Cu, (**b**) CuP-Si/Cu, (**c**) CuP-Sn/Cu, and (**d**) CuP-Zr/Cu interface structures.

Figure 11 shows a schematic of the changes in the projected density of states (PDOS) of (a) CuP/Cu, (b) CuP-Si/Cu, (c) CuP-Sn/Cu, and (d) CuP-Zr/Cu interface structures. For the CuP-Sn/Cu interface structures, see Figure 10c. It is worth noting that the Cu’s PDOS is not distinguished between Cu_3_P and Cu substrate, so they belong to the sum of Cu states in Figure 10. The energy of 3p orbital electrons of Cu overlaps with the 4d orbital electron of Sn occupying the same energy band, indicating that the electronic hybridization occurs between 3p orbital electrons of Cu overlaps with the 4d orbital electron of Sn, which enhances the bonding. For the CuP-Zr/Cu interface structures (Figure 11d), 4d orbital electrons of Zr majorly occupy the −7 eV~7 eV region, and the orbitals are relatively broad. The delocalization of 4d orbital electrons of Zr is more evident than that of 3p orbital electrons of Cu; the more substantial the delocalization effect, the better improved the conductivity and the bonding are.

## 4. Conclusions

The method used in our study is known as the first principles calculation method, which is a working theory based on density functional theory (DFT). This fruitful work explains the effects of Si, Sn, and Zr doping on the bonding strength of the CuP/Cu interface from three aspects: work of adhesion ($W_{ad}$), charge density differences, and density of states (DOS). From the result, we came to realize that:

(1) From the results, the work of adhesion ($W_{ad}$) is enhanced by adding Si, Sn, and Zr elements in the CuP/Cu interface. Since there is a significant positive correlation between the work of adhesion ($W_{ad}$) and wettability, it can be assumed that the wettability is improved by adding Si, Sn, and Zr elements. More research is warranted to ascertain further a causal relationship between the doped Si, Sn, and Zr elements in the CuP/Cu interface structure;

(2) A similar trend was observed in the charge density difference of the doped interfacial structure. We found that charge depletion around Si, Sn, and Zr atoms in the doped interfacial structure, which plays an essential role in forming Si-Cu, Sn-Cu, and Zr-Cu ionic bonds, and the charge redistribution between the interfacial atoms both become more robust than before doping. As a result, this leads to a relatively strong bond between CuP-X and Cu;

(3) The electronic hybridization occurs between the 3p orbital electrons of Cu and overlaps with the 4d orbital electron of Sn. The delocalization of 4d orbital electrons of Cu is more evident than that of 4d orbital electrons of Zr. When the delocalization is more robust, this leads to better conductivity and stronger bonding. It is reasonable to speculate that the wettability of CuP-Sn/Cu and CuP-Zr/Cu surfaces is better than the CuP/Cu surface.

There is strong evidence that doped Si, Sn, and Zr elements can effectively improve the wettability in the CuP/Cu interface structure. All of these observations are consistent with our calculated result. The advantage of first principles calculations is that they can be used to analyze charge changes and bonding at interfaces, thus, providing a deeper explanation of different interfacial microstructures. It has guiding significance for the explanation of experimental phenomena and the design of materials.

## Figures and Tables

**Table 1:** Comparison of lattice constants of Cu_3_P and Cu.

	This Work			Experiment [12,13]			Previous Calculation [14]		
	a/Å	b/Å	c/Å	a/Å	b/Å	c/Å	a/Å	b/Å	c/Å
Cu	3.621	3.621	3.621	3.620	3.620	3.620	3.621	3.621	3.621
Cu_3_P	6.974	6.974	7.199	6.959	6.959	7.143	6.960	6.960	7.180

**Table 2:** The surface energy as a function of slab thickness.

	Slab Thickness	$\gamma_{s}\;(\mathrm{J/}m^{2})$
Cu (100)	1	1.433
	2	1.448
	3	1.451
	4	1.454
	5	1.434
	6	1.433
	7	1.432
	8	1.431

**Table 3:** The formation energy of doped Si, Sn, and Zr atoms in the bulk Cu_3_P.

	The Formation Energy of Doped (eV)	
	Substituted P	Substituted Cu
CuP–Si	−1.2637	1.2642
CuP–Sn	3.4962	5.2153
CuP–Zr	3.0571	−1.1363

**Table 4:** The work of adhesion ($W_{ad}$) of the relaxed CuP-X/Cu interface.

	$\mathrm{Work}\;\mathrm{of}\;\mathrm{Adhesion}\;(W_{ad})\;(\mathrm{J/}m^{2})$
Cu_3_P/Cu	1.2869
CuP–Si/Cu	1.4273
CuP–Sn/Cu	1.3440
CuP–Zr/Cu	1.5819

## Data Availability

The data presented in this study are available in the article.
